# Targeting the Galectin Axis in Osteoarthritis: Chondroprotective Effects of Dietary and Pharmacological Phytochemicals

**DOI:** 10.3390/molecules30224391

**Published:** 2025-11-13

**Authors:** Katharina M. Pichler, Selina Kottinger, Bettina Rodriguez Molina, Jürgen Alphonsus, Sebastian Schmidt, Reinhard Windhager, Herbert Kaltner, Mario Rothbauer, Stefan Toegel

**Affiliations:** 1 Karl Chiari Lab for Orthopaedic Biology, Division of Orthopedics, Department of Orthopedics and Trauma Surgery, Medical University of Vienna, 1090 Vienna, Austria; katharina.pichler@muv.ac.at (K.M.P.); selina.kottinger@muv.ac.at (S.K.); bettina.rodriguezmolina@meduniwien.ac.at (B.R.M.); juergen.alphonsus@muv.ac.at (J.A.); reinhard.windhager@meduniwien.ac.at (R.W.); 2 Division of Orthopedics, Department of Orthopedics and Trauma Surgery, Medical University of Vienna, 1090 Vienna, Austria; 3 Chair for Biochemistry and Chemistry, Faculty of Veterinary Medicine, Ludwig-Maximilians-University Munich, 80539 Munich, Germany; sebastian.schmidt@lmu.de (S.S.); kaltner@lmu.de (H.K.); 4 Ludwig Boltzmann Institute for Arthritis and Rehabilitation, 1090 Vienna, Austria; 5 Institute of Applied Synthetic Chemistry, Faculty of Technical Chemistry, Technische Universitaet Wien, 1060 Vienna, Austria

**Keywords:** osteoarthritis, cartilage model, galectins, extracellular matrix, phytochemicals, chondroprotection, drug evaluation, translational model, spheroids

## Abstract

Background/Objectives: Galectins contribute to the pathogenesis of osteoarthritis (OA) by amplifying inflammatory and catabolic signaling, yet targeted therapeutic approaches remain limited. Three Dimensional (3D) models offer a promising platform to study human OA pathophysiology and evaluate novel interventions. Methods: We established 3D pellet cultures derived from human OA chondrocytes to investigate galectin-induced extracellular matrix (ECM) remodeling and the chondroprotective potential of phytochemicals. OA pellets were stimulated with individual galectins (Gal-1, -3, -4, -8) or a Gal-1/-3/-8 mixture, followed by co-treatment with Brazilin, Diacerein, Quercetin, Resveratrol, or Avocado-Soybean Unsaponifiables (ASU). Morphological, histological, biochemical, and gene expression analyses were performed to assess tissue integrity and molecular responses. Results: Galectin treatment induced pronounced pellet shrinkage, matrix depletion, and upregulation of matrix-degrading enzymes (MMP-1, MMP-3, MMP-13, ADAMTS-4), while suppressing matrix synthesis markers (COL2A1, COL1A1), highlighting their cooperative catabolic effects. Co-treatment with phytochemicals conferred differential protection: Brazilin and Diacerein most consistently preserved pellet size, reduced matrix-degrading gene expression, and attenuated pro-MMP-13 secretion. Resveratrol restored histological matrix density but failed to suppress pro-MMP-13 secretion. Notably, no phytochemical fully restored COL2A1 expression under galectin-induced stress. Conclusions: Our study identifies Brazilin, Diacerein, and Resveratrol as promising modulators of galectin-driven cartilage degeneration and demonstrates the translational potential of patient-derived chondrogenic pellets as a human-relevant platform for preclinical drug evaluation in OA. The 3D culture effectively recapitulates key aspects of OA pathophysiology and offers a robust system to advance therapeutic discovery targeting ECM remodeling.

## 1. Introduction

Osteoarthritis (OA) is a progressive and debilitating joint disease characterized by the breakdown of articular cartilage, synovial inflammation, and subchondral bone remodeling. It represents one of the most prevalent causes of chronic disability worldwide, with incidence and burden projected to rise in line with aging populations and increasing obesity rates. Despite its global impact, there is still no approved disease-modifying therapy, and current management strategies largely focus on alleviating symptoms rather than targeting the underlying pathophysiology [1,2]. Despite extensive research efforts, the molecular mechanisms driving OA progression remain incompletely understood, particularly the factors linking chronic inflammation to structural tissue damage. Recent studies have emphasized the importance of endogenous mediators that regulate immune responses and matrix homeostasis within the joint microenvironment [3]. Among these mediators, carbohydrate-binding proteins such as galectins have emerged as promising candidates due to their dual roles in inflammation and tissue remodeling. Several galectin family members, including Galectin-1 (Gal-1), Galectin-3 (Gal-3), Galectin-4 (Gal-4), and Galectin-8 (Gal-8), are abundantly expressed in osteoarthritic joint tissues such as cartilage [4,5,6,7], synovium and the intervertebral disc [8]. Their presence correlates with matrix degradation, activation of nuclear factor kappa B (NF-κB) signaling, and the upregulated secretion of pro-inflammatory mediators. This body of evidence suggests that galectins may serve not only as biomarkers of disease activity but also as therapeutic targets for disease modification in OA.

Targeting galectin-mediated signaling may therefore represent a novel approach to counteract OA pathogenesis. Notably, immunomodulation triggered by galectins is pharmacologically tractable, as demonstrated by synthetic inhibitors and carbohydrate-based antagonists in different disease areas [9,10]. In parallel, a wide array of naturally derived or nutritionally relevant compounds has been investigated for chondroprotective potential in OA. For instance, avocado/soybean unsaponifiables (ASU), a mixture of plant sterols and lipids, have been shown to suppress NF-κB signaling while promoting anabolic matrix synthesis via TGF-β pathways [11]. Brazilin (7,11b-dihydrobenz[b]indeno[1,2-d]pyran-3,6a,9,10(6*H*)-tetrol), a natural compound isolated from *Caesalpinia sappan* L., inhibits IL-1β-induced expression of catabolic enzymes such as matrix metalloproteinases (MMPs) and cyclooxygenase-2 (COX-2) [12]. Diacerein (4,5-diacetyloxy-9,10-dihydro-9,10-dioxo-anthracene-2-carboxylic acid), a synthetic anthraquinone derivative, attenuates IL-1β signaling and has been approved for OA treatment in some countries [13]. Furthermore, dietary polyphenols such as Quercetin (3,3′,4′,5,7-pentahydroxyflavone) and Resveratrol (3,5,4′-trihydroxy-trans-stilbene) have attracted considerable interest due to their antioxidant, anti-inflammatory, and autophagy-modulating activities. Quercetin, found in various fruits and vegetables, reduces the levels of OA-related cytokines in preclinical models [14], while Resveratrol, a stilbene primarily derived from grapes, exerts protective effects on cartilage via SIRT1 activation, NF-κB inhibition, and suppression of chondrocyte apoptosis [15]. While these agents have been extensively studied with respect to classical pro-inflammatory and catabolic cascades, their potential to modulate galectin-triggered pathomechanisms of OA has remained largely unexplored. This represents an important knowledge gap, given that galectins intersect with cytokine signaling, extracellular matrix remodeling, and apoptosis—processes directly influenced by nutritionally derived phytochemicals. Moreover, recent preclinical data with modified citrus pectin, a plant-derived galectin-3 inhibitor, highlight the feasibility of phytochemical modulation of galectins, although its effects in OA remain inconclusive [16].

Building on this rationale, the present study investigates whether selected chondroprotective compounds—including ASU, Brazilin, Diacerein, Quercetin, and Resveratrol—can modulate galectin-mediated effect in 2D and 3D cultures of human osteoarthritic chondrocytes. By elucidating the interplay between galectins and bioactive nutritional compounds, we aim to provide new insights into their roles in OA pathogenesis and to uncover novel mechanisms of action relevant for the development of future disease-modifying strategies.

## 2. Results

### 2.1. Phytochemicals Attenuate Galectin-Induced Upregulation of IL1B and MMP13 in Primary OA Chondrocytes

**Figure 1:**
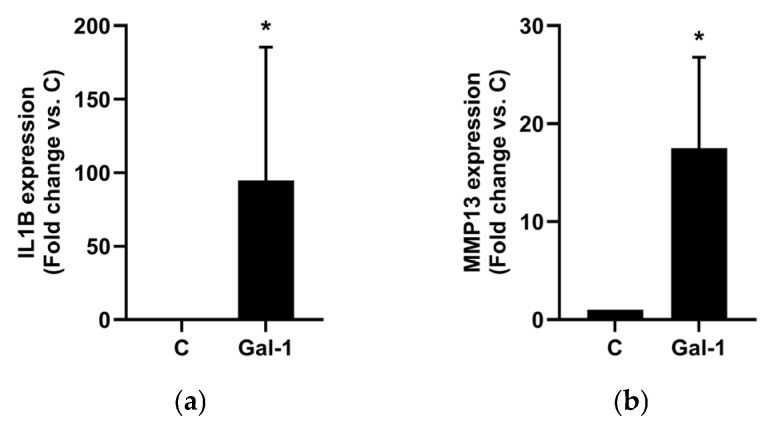
Effect of Gal-1 on OA marker expression in human chondrocytes cultured as monolayers. Using RT-qPCR, IL1B (**a**) and MMP13 (**b**) mRNA expression levels were assessed after Gal-1 treatment (10 µg/mL) and normalized to SDHA as reference gene. Shown are relative expression levels compared to untreated controls (C) set to 1 visualized as bar chart. Statistical analyses were performed using one-sided paired *t*-test (*n* = 5). Asterisks show significant *p*-values (* *p* < 0.05).

To investigate whether selected phytochemicals modulate Gal-1-mediated pro-inflammatory responses in osteoarthritic chondrocytes, we first analyzed IL1B and MMP13 mRNA expression in monolayer-cultured primary human chondrocytes isolated from five OA patients. Cells were stimulated with Gal-1 alone or in combination with ASU, Brazilin, Diacerein, Quercetin, or Resveratrol at varying concentrations. Gal-1 stimulation markedly increased IL1B (94.7 ± 90.9-fold; Figure 1a) and MMP13 (17.5 ± 9.3-fold; Figure 1b) expression compared to untreated controls.

**Figure 2:**
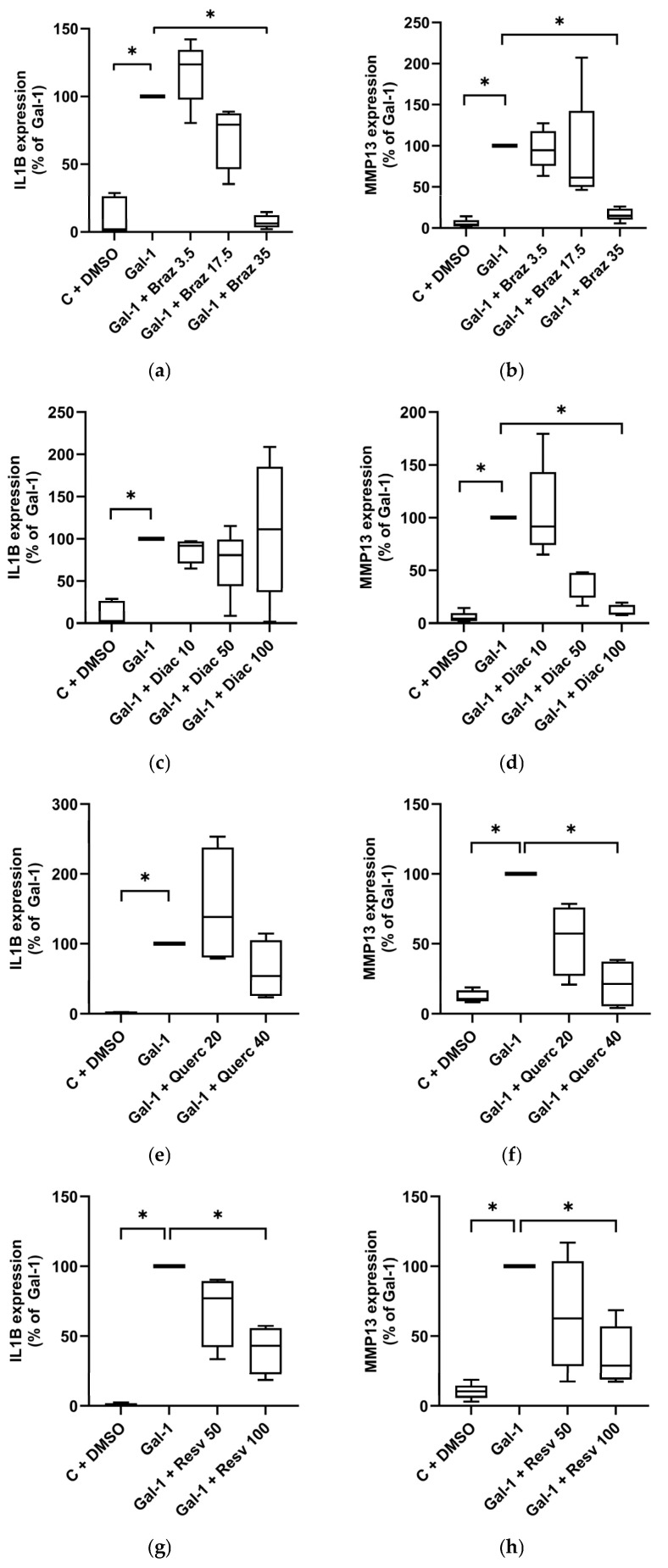
Effect of phytochemicals on OA marker expression in Gal-treated human chondrocytes cultured as monolayer. Using RT-qPCR, IL1B and MMP13 mRNA expression levels were assessed and normalized to SDHA as reference gene. Shown are relative expression levels compared to Gal-1-treated cells set to 100%. Primary chondrocytes (*n* = 5) cultured as monolayers were stimulated with Gal-1 (10 µg/mL) alone or in combination with different concentrations given in µM of Brazilin (**a**,**b**), Diacerein (**c**,**d**), Quercetin (**e**,**f**), or Resveratrol (**g**,**h**) visualized by box plots with Tukey whiskers. Statistical analyses were performed using Friedman test with pairwise comparison (**a**–**d**), or Kruskal–Wallis Test with pairwise comparison (**e**–**h**). Asterisks show significant *p*-values (* *p* < 0.05).

Co-treatment with Brazilin dose-dependently reduced the Gal-1-induced IL1B (Figure 2a) and MMP13 (Figure 2b) expression, with significant suppression to basal levels at 35 µM. Diacerein had no effect on IL1B expression (Figure 2c) but significantly decreased MMP13 levels in a dose-dependent manner, reaching baseline at 100 µM (*p* < 0.05; Figure 2d). At 40 µM, Quercetin reduced IL1B expression by 46.1% (Figure 2e), though without statistical significance, while MMP-13 was significantly reduced by 78.8% (Figure 2f). Resveratrol (100 µM) significantly suppressed both IL1B (to 43.2%; Figure 2g) and MMP13 (to 28.9%; Figure 2h). ASU had no significant effect on either marker (Figure S1a,b) expression.

**Figure 3:**
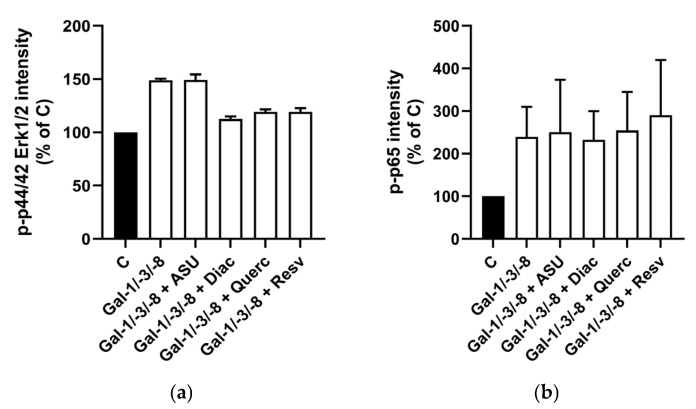
Effect of phytochemicals on signal activation in Gal-treated human chondrocytes. Bar charts with standard deviations are shown for relative signal intensities (Control set to 100%) of detected antibody stainings originating from In-Cell Western analysis (*n* = 2). Phospho-44/42 Erk 1/2 (**a**) was normalized to α-tubulin, and phospho-NF-κB (**b**) was normalized to total-NF-κB.

To explore potential molecular mechanisms, we further examined Erk1/2 and NF-κB phosphorylation in the same chondrocyte populations. We stimulated the cells with a combination of Gal-1, -3, and -8, which increased phospho-Erk1/2 by 48.9% and phospho-p65 by 139.5% (Figure 3a,b), confirming our previous findings of NF-κB activation by galectins [17]. Interestingly, co-treatment with Diacerein, Quercetin, or Resveratrol selectively reduced phospho-Erk1/2 levels—by up to 36.2% in the case of Diacerein (Figure 3a)—while phospho-p65 levels remained unchanged (Figure 3b). ASU did not alter the activation of either pathway. Together, these data indicate that, with the exception of ASU, all tested phytochemicals attenuated galectin-induced pro-inflammatory and catabolic responses in OA chondrocytes to varying extents.

### 2.2. Distinct Galectins Influence Growth, Matrix Integrity, and OA Marker Expression in Pellet Cultures

Following the observation that phytochemicals attenuated Gal-1-induced pro-inflammatory and catabolic responses in monolayer-cultured OA chondrocytes, we next evaluated the effects of distinct galectins on tissue architecture and OA-associated gene expression in a 3D cartilage model. Pellets were generated from primary human OA chondrocytes, cultured for three weeks to allow matrix deposition, and treated for two additional weeks with Gal-1, Gal-3, Gal-4, Gal-8, or a Gal-1/-3/-8 combination.

**Figure 4:**
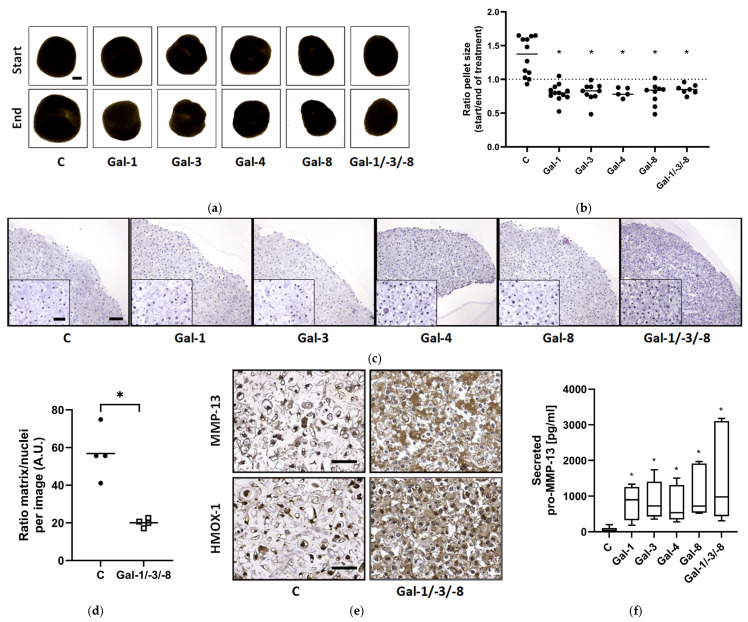
Impact of galectins on human OA cartilage-like tissues in size. OA pellets were formed within three weeks followed by two weeks of treatment with either Gal-1 (10 µg/mL), Gal-3 (18 µg/mL), Gal-4 (24 µg/mL), Gal-8 (24 µg/mL) or the mixture of Gal-1/-3/-8 (5/1/5 µg/mL). (**a**) Representative images of OA pellets from one donor at the timepoints of starting and ending the galectin treatment. Start image of control displays scale bar (500 µm). (**b**) Scatter dot plot shows the ratio of pellet sizes between the start and end after two weeks of galectin treatment. Statistical analysis was performed using Kruskal–Wallis Test with pairwise comparison. The lines indicate the medians. The dashed line represents the ratio of 1, indicating a hypothetical, stable pellet size during treatment. (**c**) Histological staining of OA pellets after two weeks of treatment, using hematoxylin and eosin. Scale bar correlates to 100 µm. (**d**) Scatter dot plot shows ratio of matrix to nuclei per image, quantified by using Image J. Each datapoint is composed of the mean of three analyzed images of controls and galectin treatment. The lines indicate the mean. For statistical analysis a *t*-test with related samples was performed (*n* = 4). (**e**) Sections of controls and galectin-treated OA pellets were stained immunohistochemically for MMP-13 and HMOX-1 (scale bar = 100 µm). (**f**) pro-MMP-13 secretion into the supernatant was measured by ELISA and shown as a box plot with Tukey whiskers. Asterisks show significant *p*-values (* *p* < 0.05).

Morphological analysis revealed that all galectin treatments reduced pellet size compared to untreated controls (Figure 4a). Quantitative assessment of the size ratio (end/start) confirmed significant growth inhibition across all treatments (Figure 4b), while untreated controls grew over time, suggesting matrix compaction or tissue shrinkage. In agreement with our previous findings [17], no cytotoxic effects of the galectins were observed. Histological examination showed reduced matrix content and disrupted cellular organization in galectin-treated pellets (Figure 4c and Figure S2). Image J (1.54g v20)-based quantification demonstrated a significant reduction in the matrix-to-nuclei ratio particularly with Gal-1/-3/-8 treatment, which significantly decreased the matrix density by 35.3% (Figure 4d). Immunohistochemistry revealed elevated protein levels of MMP-13, HMOX-1, and ADAMTS-4 after Gal-1/-3/-8 treatment (Figure 4e and Figure S3a). Control pellets exhibited weak basal cytosolic staining for MMP-13, HMOX-1, and ADAMTS-4. Following treatment with Gal-1/-3/-8 a marked increase in cytosolic staining was observed, accompanied by strong extracellular matrix staining for MMP-13 and ADAMTS-4. ELISA confirmed elevated secretion of pro-MMP-13 in response to all galectins, with the strongest effect observed for Gal-1/-3/-8 (Figure 4f).

**Figure 5:**
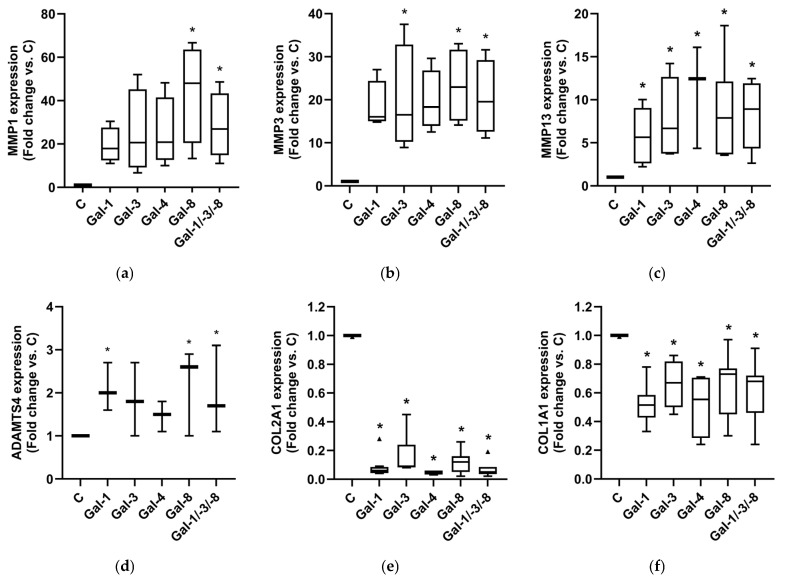
Impact of galectins on OA marker expression of human OA pellets. Relative mRNA expression of MMP1 (**a**), MMP3 (**b**), MMP13 (**c**), ADAMTS4 (**d**), COL2A1 (**e**), and COL1A1 (**f**) in OA pellets after Gal-1, Gal-3, Gal-4, Gal-8 and Gal-1/-3-/8 treatment. Untreated controls were set to 1. Kruskal–Wallis Test was performed with pairwise comparison. Triangles indicate outliers (**e**). Asterisks show significant *p*-values (* *p* < 0.05).

RT-qPCR analysis revealed strong upregulation of MMP-1 (Figure 5a), MMP-3 (Figure 5b), MMP-13 (Figure 5c), and ADAMTS-4 (Figure 5d) across all galectin treatments despite the inherent donor-dependent variability. Gal-8 and the Gal-1/-3/-8 combination consistently induced the highest expression. In parallel, the matrix-associated genes COL2A1 (Figure 5e) and COL1A1 (Figure 5f) were significantly downregulated in all galectin-treated pellets. Together, these findings indicate that individual galectins can modulate pathobiological processes in OA related to extracellular matrix remodeling. Notably, the combination of Gal-1, -3, and -8, applied at lower concentrations than the individual galectins, induced a similarly strong catabolic, matrix-degrading, and potentially pro-inflammatory phenotype in OA cartilage models. This finding suggests an additive effect of the individual galectins.

### 2.3. Phytochemicals Counteract Galectin-Induced Matrix Degradation in OA Cartilage Models

Building on these findings, we next investigated whether the catabolic and matrix-degrading effects induced by the Gal-1/-3/-8 mixture could be attenuated through co-treatment with selected phytochemicals, using the same 3D OA cartilage model. The chondroprotective activity of the phytochemicals was assessed in terms of morphological preservation, protein secretion, and gene expression changes.

**Figure 6:**
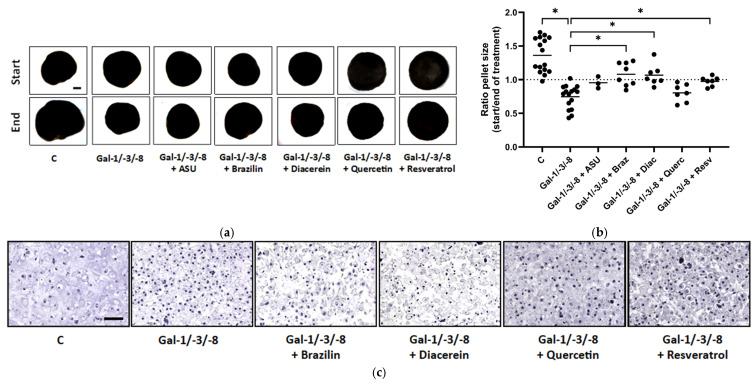

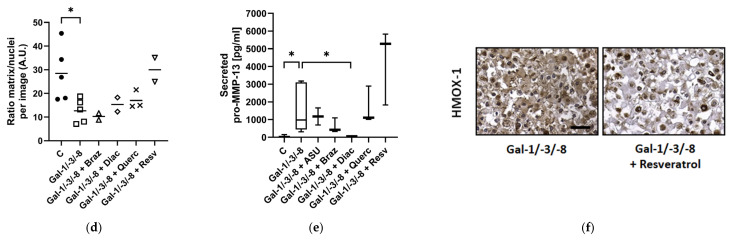
Phytochemicals counteract galectin-induced matrix degradation in OA pellets. (**a**) Representative images of OA pellets from one donor at the start and end of the two-week treatment period. Pellets were pre-cultured for three weeks before treatment with either a galectin mixture (Gal-1/-3/-8 at 5/1/5 µg/mL) alone or in combination with one of the following phytochemicals: ASU (40 µg/mL; Gal-1/-3/-8 + ASU), Brazilin (35 µM; Gal-1/-3/-8 + Braz), Diacerein (50 µM; Gal-1/-3/-8 + Diac), Quercetin (40 µM; Gal-1/-3/-8 + Querc), or Resveratrol (100 µM; Gal-1/-3/-8 + Resv). Scale bar in the control start image equals 500 µm. (**b**) Scatter dot plot showing the size ratio of individual pellets (end/start) after two weeks of treatment. The dashed line indicates a ratio of 1, representing unchanged pellet size. Statistical analysis was performed using the Kruskal–Wallis test with pairwise comparisons; lines indicate medians. (**c**) Histological analysis of OA pellets after two weeks of treatment using hematoxylin and eosin (H&E) staining. Scale bar: 100 µm. (**d**) Scatter dot plot shows ratio of matrix to nuclei per image, quantified by using Image J. Each datapoint represents the mean of three analyzed images of each condition. The lines indicate medians. (**e**) ELISA quantification of secreted pro-MMP-13 in the culture supernatant shown as box plots with Tukey whiskers. (**f**) Immunohistochemical staining of pellet sections for HMOX-1. Scale bar: 100 µm. Asterisks show significant *p*-values (* *p* < 0.05).

Quantitative analysis of pellet size dynamics, calculated as the ratio of pellet size at the end versus the start of the treatment period, revealed diverse effects of phytochemical co-treatment (Figure 6a,b). While Gal-1/-3/-8 treatment led to marked pellet shrinkage, co-treatment with Brazilin or Diacerein significantly preserved pellet size and even promoted mild pellet growth over time (median ratios exceeding 1.0), suggesting substantial chondroprotective potential. Histological evaluation by H&E staining revealed pronounced structural degradation in galectin-treated pellets, characterized by reduced matrix density and disrupted architecture (Figure 6c). Phytochemical co-treatments mitigated these degenerative changes to varying degrees. Quantitative image analysis of histological sections revealed that Quercetin and Resveratrol elicited the most robust restoration of matrix-to-nuclei density (Figure 6d), which reflects improved structural preservation. To assess matrix-degrading enzyme activity, secreted pro-MMP-13 levels were quantified by ELISA (Figure 6e). Galectin treatment significantly elevated pro-MMP-13 secretion from 26.2 pg/mL to 977 pg/mL (as compared to the median of the controls), confirming strong catabolic activity. Co-treatment with Diacerein fully restored pro-MMP-13 levels, underscoring its potent anti-catabolic effects. In contrast, Resveratrol failed to suppress Gal-1/-3/-8 induced pro-MMP-13 secretion, although it improved matrix structure as demonstrated by histological analysis. Immunohistochemical staining for HMOX-1, a marker for cellular stress, revealed reduced cytosolic expression in pellets treated with Gal-1/-3/-8 and Resveratrol (Figure 6f).

**Figure 7:**
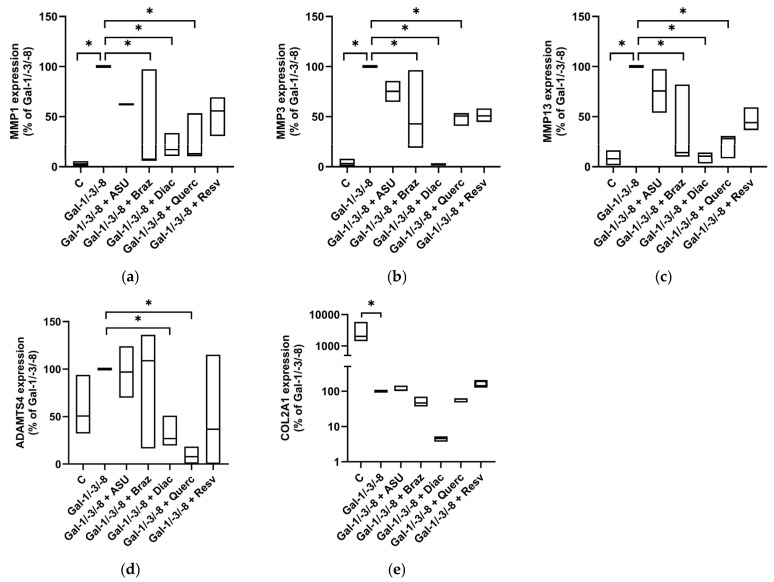
Impact of phytochemicals on OA marker expression of OA pellets. Relative mRNA expression levels in percent (Gal-1/-3/-8 was set to 100%) shown as floating bar plots (line at median) for MMP1 (**a**), MMP3 (**b**), MMP13 (**c**), ADAMTS4 (**d**), and COL2A1 (**e**). Statistical significance was determined by Kruskal–Wallis test with pairwise comparisons. Asterisks indicate statistically significant differences (* *p* < 0.05).

To corroborate these protein-level data, we next assessed the mRNA expression of key matrix-degrading enzymes and cartilage matrix components by qPCR. As expected, Gal-1/-3/-8 treatment significantly upregulated MMP1, MMP3, MMP13, and ADAMTS4, while downregulating COL2A1 expression (Figure 7a–e). Co-treatment with Brazilin, Diacerein and Quercetin markedly reduced MMP1 (Figure 7a), MMP3 (Figure 7b), MMP13 (Figure 7c), and ADAMTS4 (Figure 7d) expression, while ASU exhibited only modest effects. Importantly, none of the tested phytochemicals was able to restore Gal-1/-3/-8-supressed COL2A1 mRNA levels (Figure 7e). Collectively, these data demonstrate that phytochemicals confer differential but substantial protection against galectin-induced matrix degradation in an OA cartilage model. Among the tested compounds, Brazilin, Diacerein, and Resveratrol showed the most consistent chondroprotective effects across multiple complementary readouts.

## 3. Discussion

Osteoarthritis (OA) remains a major cause of disability worldwide, driven by an interplay of inflammatory processes and extracellular matrix (ECM) degradation [1]. While current treatments focus on symptomatic relief, disease-modifying therapies capable of halting or reversing cartilage degeneration are still lacking. Prior work from our group has identified Gal-1, Gal-3, Gal-4, and Gal-8 as potent modulators of chondrocyte activity and matrix turnover, amplifying both inflammatory and catabolic signaling cascades in OA cartilage [4,5,6,7]. Consistent with earlier reports, Gal-1 stimulation in 2D chondrocytes strongly induced IL1B and MMP13 expression, effects that were synergistically enhanced when Gal-1 was combined with Gal-3 and Gal-8. These findings underscore the capacity of multiple galectins to cooperatively activate NF-κB and Erk1/2 signaling and to act as upstream amplifiers of pro-inflammatory and catabolic pathways. Notably, the observed lack of galectin regulation at the mRNA and protein level confirms that these molecules act predominantly as upstream amplifiers of inflammatory responses rather than being downstream targets themselves [4].

In the present study, we extended these insights using a translationally relevant 3D human cartilage model, which more closely mirrors native tissue architecture than conventional monolayer cultures. Despite their higher cost and lower treatment sensitivity, 3D cultures offer a more physiologically relevant setting in respect of cell–cell communication, cell–matrix interaction and cell differentiation [18,19,20]. In pellet cultures, galectin exposure led to tissue shrinkage, matrix disorganization, upregulation of MMPs (MMP1, -3, -13) and ADAMTS4, and concomitant suppression of COL2A1. Together, these data confirm that galectins not only trigger inflammatory signaling but also directly drive structural ECM breakdown, positioning them as key upstream effectors in OA progression. To our knowledge, this is the first study to systematically investigate whether dietary and pharmacological phytochemicals with known chondroprotective properties can modulate galectin-induced effects in human OA chondrocytes. The tested substances—Brazilin [21], Diacerein [22], Resveratrol [23], Quercetin [14], and Avocado/Soy Unsaponifiables [11]—differ in structure but share certain functional characteristics. Brazilin (a homoisoflavonoid), Resveratrol (a stilbenoid), and Quercetin (a flavonoid) all contain aromatic rings and multiple hydroxyl groups, conferring antioxidant and radical-scavenging properties. Diacerein, an anthraquinone derivative, contains both keto and hydroxyl groups, while ASU represents a complex mixture of plant-derived sterols and other lipophilic compounds. These functional groups are generally associated with antioxidant and anti-inflammatory activities and have been widely studied in OA models. In our experiments Brazilin, Diacerein, and Resveratrol effectively reduced galectin-mediated effects in chondrocytes monolayer, most likely by modulating downstream signaling cascades such as NF-κB and MAPK. In contrast, Quercetin and ASU, although relevant in OA research, did not significantly attenuate galectin-induced responses under our experimental conditions. Importantly, in 3D pellets, Brazilin and Diacerein not only attenuated matrix-degrading enzyme expression but also preserved or even promoted pellet growth, suggesting broader tissue-stabilizing properties that go beyond simple anti-catabolic effects. Resveratrol most effectively restored matrix density but failed to normalize MMP-13 expression and pro-MMP-13 secretion, which was shown by [24] after treating IL-1β stimulated human OA articular chondrocytes with Resveratrol in a dose-dependent manner. The inability of any compound to fully restore COL2A1 expression suggests that phytochemicals primarily stabilize existing matrix integrity rather than initiating de novo matrix synthesis under galectin-induced stress. Given the high specificity of the galectin carbohydrate recognition domain (CRD) for galactose-like structures [25], direct binding of these compounds to galectins appears unlikely. Instead, their effects are more plausibly explained by the inhibition of pro-inflammatory and pro-apoptotic signaling, including pathways involving NF-κB, MAPK and Caspase-3. The shared capacity of Brazilin, Diacerein, and Resveratrol to suppress MMP expression, inflammation, and apoptosis likely accounts for their ability to counteract galectin-induced osteoarthritic phenotypes.

From a translational standpoint, these findings support a two-tiered perspective: First, galectins emerge as attractive upstream targets whose inhibition may simultaneously dampen inflammation and prevent matrix breakdown. Second, phytochemicals such as Brazilin and Diacerein offer promising avenues for adjunctive OA therapy by stabilizing cartilage integrity and counteracting galectin-driven catabolic cascades. The use of a 3D human cartilage model in this study underscores the feasibility of translating such approaches into clinically relevant tissue environments, bridging the gap between molecular insights and therapeutic application.

## 4. Materials and Methods

### 4.1. Chondrocyte Isolation and Cell Culture

Human articular cartilage samples were collected from OA patients undergoing total knee arthroplasty, following written informed consent and approval by the Ethics Committee of the Medical University of Vienna (EK-No. 1822/2017 and 1555/2019). OA chondrocytes were isolated from the femoral condyles and tibial plateaus and seeded as monolayer in growth medium (DMEM containing 4.5 g/L D-Glucose, 25 mM HEPES (Gibco, Thermo Fisher Scientific, Inc., Waltham, MA, USA)), further supplemented with 10% Fetal Bovine Serum (FBS) South America (Biowest, Nuaillé, France) and 1% Antibiotic-Antimycotic (Gibco)). To preserve their native phenotype, only primary chondrocytes without passaging were used in all experiments. Cells were grown at 37 °C in 5% CO_2_. Once monolayer cultures reached approximately 90% confluency, cells were serum-starved overnight and subsequently stimulated. To establish 3D pellet cultures, 4 × 10^5^ OA chondrocytes were seeded into 1.5 mL tubes in growth medium and centrifuged at 1000 rpm for 10 min at room temperature. The centrifuged cells were cultivated for two days in growth medium which then was changed to starvation medium (DMEM containing 4.5 g/L D-Glucose, 25 mM HEPES (Gibco), further supplemented with 1% Antibiotic-Antimycotic (Gibco) and 1% ITS (Gibco)). Following formation for three weeks at 37 °C in 5% CO_2_, pellets were pre-treated with single phytochemicals for 48 h and then treated with galectins in presence or absence of the phytochemicals for two weeks prior to mRNA isolation or processing for histologic examination. Treatments in fresh starvation medium were renewed two times per week. Controls were treated with DMSO or ethanol, depending on the solvent of the phytochemical. Supernatants of the pellets were collected for ELISA analysis. The pellets were visualized with Nikon ECLIPSE TE2000-U (Nishi-Ōi, Shinagawa, Tokyo). The analysis was performed with the software NIS-Elements (Version 6.10.01) whereby the pellet diameter was measured and expressed in mm. The area of the pellet was calculated with the formula A = (π/4) × d^2^.

### 4.2. Galectins

For recombinant protein expression of galectins, *E. coli* BL21 (DE3) pLysS cells were transformed with the pGEMEX-1 (Promega, Walldorf, Germany) plasmids carrying the coding sequences of Gal-1, -3, -4, or -8. A colony of the transformed bacteria was grown for 16 h at 37 °C in Luria Broth (LB) medium (100 mL) containing the antibiotic ampicillin for selection. For protein expression, 5 mL of the bacterial LB suspension was given to 1 L Terrific Broth (TB) medium containing ampicillin. After an initial growth for 2–3 h at 37 °C in TB medium up to an OD at 600 nm around 0.6, protein expression was induced using 100 μM β-1-thio-d-galactopyranoside (IPTG), and bacteria were cultured at 37 °C for further 16 h. Cells were harvested and washed, and bacterial pellets were frozen for 2 h at −20 °C before being lysed by sonication at 4 °C. The protein was purified from the bacterial extracts after lysis by affinity chromatography on lactosylated Sepharose 4 B [26]. Active protein was eluted with 50 mM lactose in 20 mM PBS, pH 7.2, followed by buffer exchange to 10 mM PBS, pH 7.2, using a PD10 column (Merck, Taufkirchen, Germany) to remove lactose. Purity was ascertained by one-dimensional gel electrophoresis under denaturing conditions. Additionally, activity was tested by hemagglutination as described previously [27].

For treatment of pellet cultures, the combination of Gal-1, Gal-3, and Gal-8 is referred to Gal-1/-3/-8. Differences in the concentrations of galectins were deliberately chosen to adjust for differences in their molecular weight. Following concentrations of galectins were used: Gal-1 (10 µg/mL), Gal-3 (18 µg/mL), Gal-4 (24 µg/mL), Gal-8 (24 µg/mL) or the mixture of Gal-1/-3/-8 (5/1/5 µg/mL).

### 4.3. Anti-Inflammatory Compounds

Avocado Soybean Unsaponifiables (ASU) from Laboratoires Expanscience (Paris La Défense, France) was applied with 20 or 40 µg/mL, Brazilin (Braz) a kind gift from [28] with 3.5, 17.5, or 35 µM, purity ≥ 95% (HPLC); Diacerein (Diac 50 or 100 µM; Sigma-Aldrich (St. Louis, MO, USA), D9302, purity ≥ 95% (HPLC)), Quercetin (Querc 20 or 40 µM; Biosynth (Staad, Switzerland), FQ32160, >97% (HPLC)), and Resveratrol (Resv 50 or 100 µM; Sigma-Aldrich, R5010, trans-isomer, purity ≥ 99% (HPLC)). ASU was dissolved in ethanol, while Brazilin, Diacerein, Quercetin, and Resveratrol, were solubilized in dimethyl sulfoxide (DMSO). Concentrations were selected based on published data [11,15,22,29,30]. All substances were applied to serum-starved OA chondrocyte cultures solely or in the presence of Gal-1 (10 µg/mL) in 2D experiments or Gal-1/-3/-8 (5/1/5 µg/mL) in 2D and 3D experiments.

### 4.4. RT-qPCR

Total RNA isolation, cDNA synthesis, and SYBR Green-based quantitative real-time PCR (qPCR) were performed as previously described [4]. In brief, total RNA was extracted using the innuPREP RNA Mini Kit (IST Innuscreen GmbH, Reinach, Switzerland), and RNA purity and concentration were assessed with the NanoDrop 2000 spectrophotometer (Thermo Fisher Scientific, Inc.). Reverse transcription was carried out using standard protocols. Relative mRNA expression levels were calculated using amplification efficiencies and were normalized to the expression of succinate dehydrogenase complex subunit A (SDHA), which was validated as a stable reference gene under the experimental conditions applied in this study.

### 4.5. In-Cell Western (ICW) Assay

OA chondrocytes were cultured in 96-well plates until they reached full confluency. After serum starvation, cells were pre-treated with phytochemical compounds as follows: ASU (40 µg/mL), Diacerein (50 µM), Quercetin (40 µM) and Resveratrol (100 µM) for 15 min, followed by stimulation with Gal-1/-3/-8 (5/1/5 µg/mL) for 60 min. Subsequently, cells were washed with PBS and fixed, either with cold methanol (−20 °C) for 10 min at room temperature, or 4% freshly prepared paraformaldehyde for 10 min at room temperature, depending on the antibodies. After removal of the fixative agent, cells were washed with PBS and paraformaldehyde-fixed cells were additionally permeabilized with 0.1% Triton-X. Then, cells were washed and blocked using LI-COR Odyssey blocking buffer for at least 90 min at room temperature with gentle agitation. Primary antibodies were applied overnight at 4 °C in blocking buffer: anti-NF-κB p65 (mouse, Cell Signaling Technology (Danvers, MA, USA), #6956S, 1:1000) and anti-phospho-NF-κB p65 (Ser536) (rabbit, Cell Signaling, #3033S, 1:800) or anti-α-tubulin (mouse, Cell Signaling, 3873S, 1:800) and anti-phospho-p44/42 MAPK Erk1/2 (Thr202/Tyr204) (rabbit, Cell Signaling, 9101S, 1:500). On the following day, cells were washed with PBS containing 0.1% Tween 20 and incubated for 1 h at room temperature with secondary antibodies diluted in blocking buffer containing 0.2% Tween 20, donkey anti-mouse IgG IRDye™ 680RD (LI-COR Biosciences (Lincoln, NE, USA), 1:1000) and goat anti-rabbit IgG IRDye™ 800CW (LI-COR Biosciences, 1:1000). After incubation, cells were washed with PBS/Tween 20 (0.1% *v*/*v*) and the plates were air-dried and scanned using the Odyssey CLx Infrared Imaging System (LI-COR Biosciences). The signal intensity was read out using Image Studio software (Version 5.2, LI-COR Biosciences) and phosphorylated proteins were normalized either to total-NF-κB or α-tubulin. 

### 4.6. Histology and Immunohistochemistry

Pellets were processed according to standard histology procedures [17]. Paraffin sections (2.5 μm thickness) were stained with HE and immunohistochemically using anti-MMP-13 (mouse; R&D Systems (Minneapolis, MN, USA), MAB 511, 1:50), anti-ADAMTS-4 (rabbit; Sigma-Aldrich, A4976, 1:500) and anti-HMOX-1 (rabbit; Abcam (Cambridge, UK), ab13243, 1:100). Negative controls were performed without primary antibody (Figure S3b). To quantify the matrix/nuclei ratio, three HE images were taken randomly of each pellet per condition. The images were split by Image J (Image J 1.54g v20) in three channels (red, green and blue), and the red channel was used to detect the area fraction of matrix and nuclei with distinct threshold settings. The signal intensity of the matrix was normalized to the signal intensity of nuclei. HE images were visualized with an Olympus SC-50 camera (Tokyo, Japan) with the Olympus cellSens Entry software (Version 3.2).

### 4.7. Enzyme-Linked Immunosorbent Assay (ELISA)

Cell culture supernatants were collected from untreated pellets and pellets treated either with Gal-1, Gal-3, Gal-4, Gal-8 solely or in combination with Gal-1/-3/-8 in presence or absence of ASU, Brazilin, Diacerein, Quercetin or Resveratrol, prior to centrifugation and storage at −80 °C. The concentration of Pro-MMP-13 was determined using a quantitative ELISA according to the manufacturer’s instructions (R&D Systems, Minneapolis, MN, USA). Briefly, microplates pre-coated with a monoclonal antibody specific for human Pro-MMP-13 were incubated with standards and samples, allowing the antigen to bind to the immobilized capture antibody. After washing to remove unbound material, an enzyme-linked monoclonal detection antibody specific for human Pro-MMP-13 was added. Following an additional wash step, a substrate solution was applied, resulting in a colorimetric reaction proportional to the amount of bound Pro-MMP-13. The reaction was terminated, and absorbance was measured using a microplate reader. The standard curve ranged from 78 to 5000 pg/mL.

### 4.8. Statistics

All experiments were repeated independently with primary chondrocytes from different OA patients. Statistical analyses were performed using SPSS 29.0. Normal distribution of the data was analyzed using the Shapiro–Wilk test. Statistical significance of normally distributed data underwent a paired *t* test. For non-normally distributed data with related samples, the Friedman test was conducted, whereas for independent groups or groups with missing datapoints, the Kruskal–Wallis Test with pairwise comparison was used. *p*-Values of less than 0.05 were considered significant (* *p* < 0.05).

## 5. Conclusions

In summary, our study identifies galectins as potent amplifiers of inflammatory and catabolic signaling in OA and demonstrates that selected phytochemicals—particularly Brazilin, Diacerein, Quercetin, and Resveratrol—can counteract galectin-induced matrix degradation in a human 3D cartilage model. These findings highlight galectins as promising therapeutic targets and position phytochemicals as candidate agents for nutritionally inspired disease-modifying strategies in OA. By uncovering an unexplored intersection between galectin biology and phytochemical action, this work opens new avenues for combination therapies that integrate galectin inhibition with pro-anabolic interventions to better preserve cartilage integrity.

## Data Availability

The original contributions presented in this study are included in the article and Supplementary Materials, further inquiries can be directed to the corresponding author.

## Supplementary Materials

The following supporting information can be downloaded at: https://www.mdpi.com/article/10.3390/molecules30224391/s1, Figure S1: Impact of ASU on OA marker expression of human OA chondrocytes; Figure S2: Matrix and nuclei ratio after galectin treatment of OA pellets; Figure S3: Immunohistochemical staining of OA pellets with ADAMTS-4.
