# Supplementary material for: Targeting the Galectin Axis in Osteoarthritis: Chondroprotective Effects of Dietary and Pharmacological Phytochemicals
Source: Molecules. 2025 Nov 13;30(22):4391. doi: 10.3390/molecules30224391 (PMC12655804; doi:10.3390/molecules30224391)
Supplement: Supplementary file 1 [file molecules-30-04391-s001.zip › molecules-3869835-supplementary.pdf]

Supplemental Figure S1

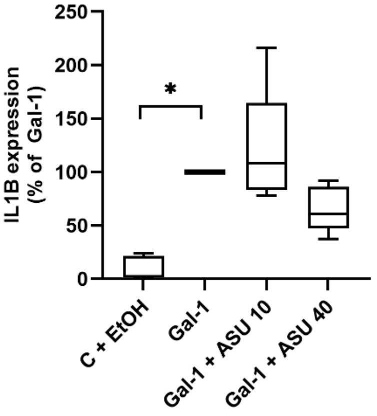

(**a**)

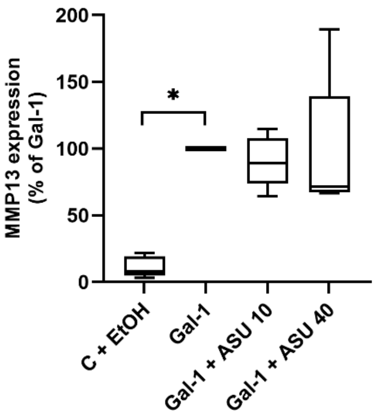

(**b**)

Supplemental Figure S2

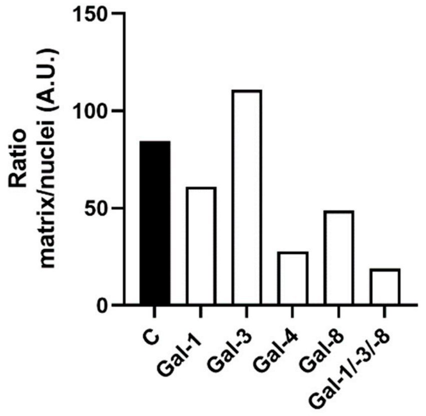

Supplemental Figure S3

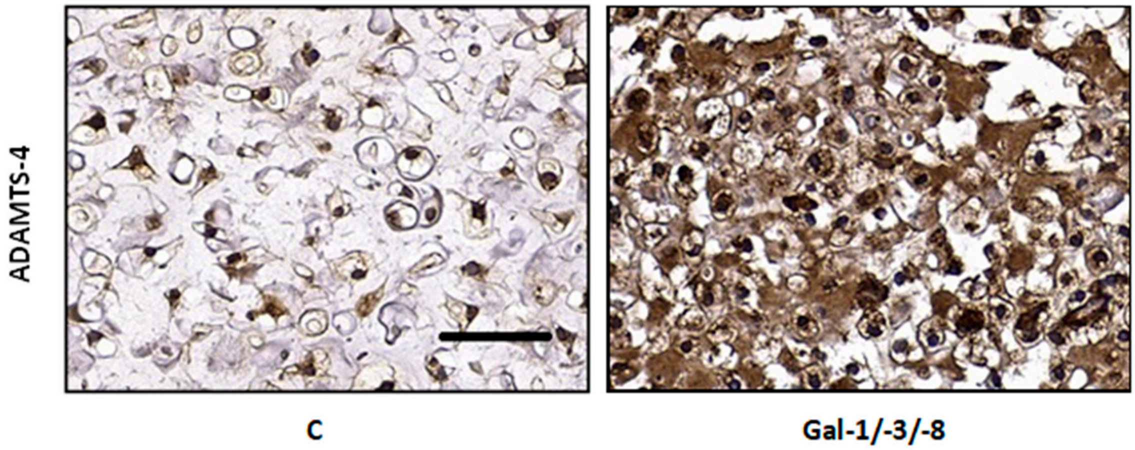

(**a**)

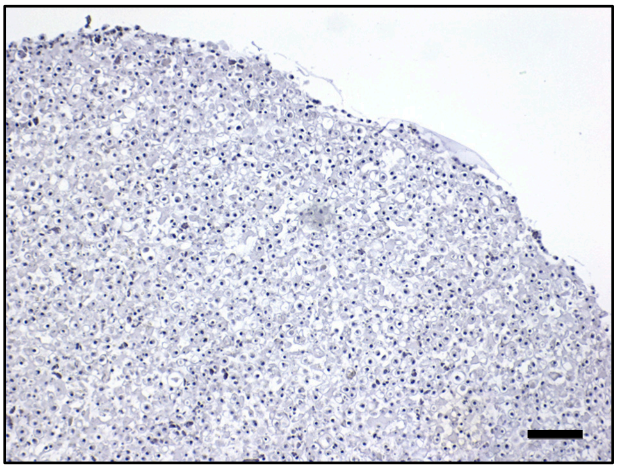

Neg. Ctrl

(**b**)

Supplemental Figure Legend

Figure S1. Impact of ASU on OA marker expression of human OA chondrocytes. (**a**,**b**) Box plots with Tukey whiskers show mRNA expression levels of IL1B (**a**) and MMP13 (**b**) set to 100% of Gal-1-treated cells with different ASU concentrations. Genes

were normalized to SDHA as reference gene. Asterisks show significant p-values (*p°<°0.05)

Figure S2. Matrix and nuclei ratio after galectin treatment of OA pellets.

(**a**) Staining intensity of matrix and nuclei were quantified by Image J of 20-fold magnified microscopic HE pictures of control and galectin-treated OA pellets. Bar chart shows the ratio of matrix and nuclei of one donor. Asterisks show significant p-values (*p°<°0.05)

Figure S3. Immunohistochemical staining of OA pellets with ADAMTS-4

(**a**) Sections of controls and galectin-treated OA pellets were stained immunohistochemically for ADAMTS-4 (scale bar equals 100 μm). (**b**) OA pellet treated with Gal-1/-3/-8 and Quercetin stained immunohistochemically without primary antibody (scale bar equals 100 μm), indicating the negative control.
